
JOURNAL INFORMATION
==============================
NLM Title Abbreviation: ScientificWorldJournal
Journal ID: ScientificWorldJournal
NLM Journal ID: 101131163
Journal ID: TSWJ

The Scientific World Journal

ISSN: 2356-6140
EISSN: 1537-744X
Publisher: Wiley

ARTICLE INFORMATION
==============================
PMCID: PMC6084223
PMID: 30147604
DOI: 10.1100/tsw.2001.216
Article ID: 739129
Article version: 1
Subjects: Short Report

Identification and Sequencing of Bcl and Apaf1 Genes in the Common Carp, Cyprinus Carpio

Chakravarthy C. *
Williams G.T.
Hoole D.
School of Life Sciences, Huxley Building, Keele University, Staffordshire ST5 5BG, UK
*C. Chakravarthy: c.chakravarthy@biol.keele.ac.u
Publication date: 2001
Electronic publication date: 2001 Feb 9
Volume: 1
First page: 123
Last page: 123
Copyright: Copyright © 2001 C. Chakravarthy et al.
Copyright year: 2001
License: This is an open access article distributed under the Creative Commons Attribution License, which permits unrestricted use, distribution, and reproduction in any medium, provided the original work is properly cited.
License URL: https://creativecommons.org/licenses/by/3.0/

==============================
Miami *Nature Biotechnology* Short Reports
*TheScientificWorld* (2001) 1 (S3), 123SR
ISSN 1532-2246; DOI 10.1100/tsw.2001.216

### IDENTIFICATION AND SEQUENCING OF BCL AND APAF1 GENES IN THE COMMON CARP, *Cyprinus carpio*

C. Chakravarthy*, G.T. Williams, D. Hoole

School of Life Sciences, Huxley Building, Keele University, Staffordshire ST5 5BG U.K.
* c.chakravarthy@biol.keele.ac.u

**INTRODUCTION.** The role of apoptosis in the immune system is being studied in cultured carp, *Cyprinus carpio* an economically important farmed fish. In intensive culturing systems, stress is suspected of reducing the immune competence of farm animals. Our initial studies investigated the effect of dexamethasone, a corticosteroid analogue, on the different immunocompetent cells in the pronephros and thymus. This is currently being extended to identify and sequence an apoptotic regulator (Bcl-x) and an apoptotic activator gene (APAF1) from the carp cDNA library.

**METHOD.** Cells from the pronephros and thymus were maintained *in vitro*, in L-15 medium (Sigma) supplemented with 2mM L- glutamine and containing penicillin (100i.u./ml), streptomycin (100μg/ml), and 5% heat- inactivated foetal calf serum, at 20$^{o}$C. They were then exposed to different concentrations of dexamethasone (0.1μM to10.0μM$^{2}$) for up to 48h in time. Control cells were those maintained in the culture medium lacking in dexamethasone. Cell viability and percentage of apoptotic cell death at times 0h, 24h and 48h were determined using nigrosine dye exclusion and acridine orange stain respectively. DNA from approximately $10^{6}$ cells was isolated from each of the above samples and subjected to agarose gel electrophoresis.

In the differential experiment, the different types of immunocompetent cells were labelled with monoclonal antibodies WCI04, WCI09 and WCI12 and their responses to 1.0μM dexamethasone over 6 hours was recorded.

Degenerate primers for the Bcl-xL equivalent in carp were designed following a homology study on the sequences of *Homo sapiens* (human) and *Xenopus laevis* (South African clawed frog). The homology between human APAF1 and that of *Danio rerio* (Zebrafish) was examined, and primers constructed, for the comparable sequence in the carp. These were used in PCR to obtain sequences from the carp cDNA library. The sequences obtained will now be used as probes to sequence the rest of the gene(s).

**RESULTS.** Statistical analysis reveals that there is a significant difference between the number of control cells undergoing apoptotic cell death and that of cells exposed to the different concentrations of dexamethasone ($p<0.001$) (Fig.1). This effect is greatest between the 0h and 24h time periods and appears to decrease after this time period, and is similar in the pronephros and the thymus.

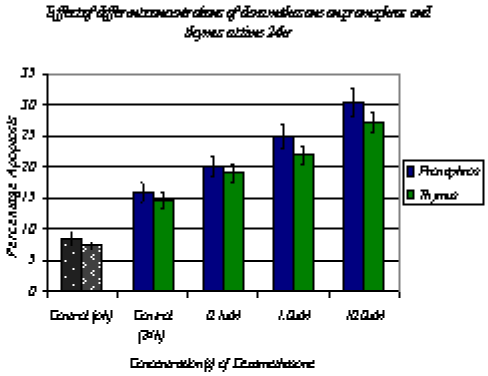

*Figure1 Graph showing the effect of apoptosis at 24h after exposure to dexamethasone*

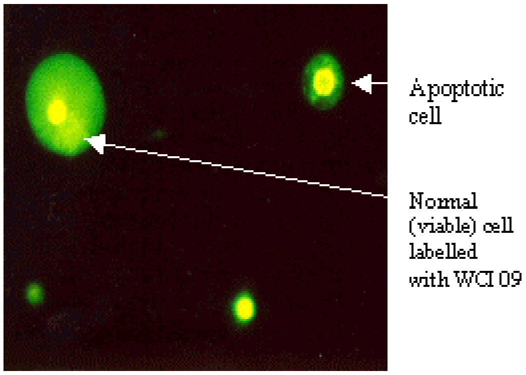

*Figure 2 Cells stained with acridine orange*

Studies using monoclonal antibodies that recognised various surface components of carp leukocytes revealed that the thymocytes are more susceptible to apoptotic death induced by dexamethasone, when compared to the B-cells.

The initial sequence obtained from the carp cDNA library suggests that the isolate has a close homology to the zebrafish APAF1. Results of the Bcl-xL gene are swaited.

**DISCUSSION.** Dexamethasone is an analogue of cortisol, a stress hormone that is known to induce apoptosis. These studies have established the fact that dexamethasone induces apoptosis in the immune cells of pronephros and thymus and also that the response is differential, in the various cells. When the complete gene sequences are confirmed, the effect of cortisol on the expression of Bcl-x and APAF1 will be ascertained. This would lead to an understanding of how the stress hormone affects the primary response of the immune system[1] at the genetic level.

**ACKNOWLEDGEMENT.** The monoclonal antibodies were kindly given to us by Dr. J. Rombout, Wanginghen University.

**REFERENCES.**

1. Clem, L.W., Bly, J.E., Ellaesser, C.F., Lobb, C.J., and Miller, N.W. (1990) J. Exp. Zool. 4, 123-125
2. Unpublished results of B. Swain, D. Hoole, and G.T. Williams, School of Life Sciences, Keele University, U.K.
